# GSK-J4 Suppresses Tumorigenesis by Targeting the PERK-c-Myc Pathway Through Endoplasmic Reticulum Stress Activation in Tuberous Sclerosis Complex

**DOI:** 10.3390/ijms27073067

**Published:** 2026-03-27

**Authors:** Xin Lei, Tao Lang, Ping Li, Changxin Wu

**Affiliations:** 1 Shanxi Provincial Key Laboratory of Medical Molecular Cell Biology, Institutes of Biomedical Sciences, Shanxi University, Taiyuan 030006, China; leejunkilx@sina.com (X.L.);; 2 Key Laboratory of Chemical Biology and Molecular Engineering of Ministry of Education of China, Shanxi University, Taiyuan 030006, China

**Keywords:** tuberous sclerosis complex, histone modification, rapamycin, GSK-J4, apoptosis, endoplasmic reticulum stress

## Abstract

The limited and inconsistent efficacy of existing therapies for tuberous sclerosis complex (TSC) has driven the exploration of novel strategies, including epigenetic regulation. GSK-J4, an inducer of global H3K27me3 accumulation, shows broad anti-tumor activity. However, its therapeutic potential in TSC remains unclear. In the study, we reported that GSK-J4 inhibited cell cycle progression and induced apoptosis in primary *Tsc1^+/−^* and *Tsc2^+/−^* MEFs. Mechanistically, *Tsc1* or *Tsc2* deletion reduced global H3K27me3, correlating with increased viability, accelerated cell cycle, and suppressed apoptosis-phenotypes reversed by GSK-J4. Moreover, GSK-J4 triggered endoplasmic reticulum stress (ERS) by activating the PERK-ATF4-CHOP axis, which concurrently downregulated the proto-oncogene c-Myc, outlining a GSK-J4→p-PERK→c-Myc inhibitory pathway. Notably, GSK-J4 synergized with rapamycin to enhance cell cycle arrest and apoptosis. *In vivo*, this combination alleviated renal impairment in *Tsc1*- or *Tsc2*-deficient models, suggesting a promising therapeutic strategy for TSC patients with suboptimal response to mammalian target of rapamycin complex 1 (mTORC1) inhibitors. Our study elucidates a specific ERS-dependent anti-tumor mechanism of GSK-J4 in *Tsc*-deficient contexts and demonstrates the synergistic efficacy of combining epigenetic and mTORC1 inhibitors.

## 1. Introduction

Tuberous sclerosis complex (TSC), characterized by germline mutations in either *TSC1* or *TSC2*, manifests as multi-organ tumors due to the constitutive mammalian target of rapamycin complex 1 (mTORC1) hyper-activation [1]. Renal angiomyolipomas and neurological pathologies dominate the clinical presentations. Current treatment guidelines recommend mTORC1 inhibitors (e.g., everolimus, sirolimus) as first-line therapy [2,3]. However, mTORC1 inhibitors face three intrinsic limitations: (i) treatment-resistant tumor recurrence post-cessation [4,5], (ii) dose-dependent adverse effects [6,7,8,9] (e.g., stomatitis, immunosuppression), and (iii) interpatient efficacy variability. These challenges underscore the critical need for novel therapeutic strategies for TSC patients.

Epigenetic dysregulation has emerged as a key contributor to tumorigenesis [10,11,12]. For instance, dysregulation of the methylation of H3K27, which is dynamically modulated by the lysine methyltransferase (EZH2) [13] and demethylases (KDM6A and KDM6B) [14], has been described in a number of pathological conditions, including cancer. Recently, the loss of H3K27me3 has been shown to occur in radiation-induced meningiomas, and the extent of this loss is positively correlated with prevalence and recurrence rates [15]. In hematological malignancies, such as lymphomas, EZH2, which mediates the trimethylation of H3K27, is at high-expression levels [16]. Although abnormal epigenomic characteristics can regulate cell function and contribute to cancer development, such alterations may also be countered through the use of epigenetic drugs [17]. 5-Azacitidine, a DNA methylation inhibitor and the first epigenetic drug approved by the U.S. FDA, is used to treat myelodysplastic syndromes (MDS) and acute myeloid leukemia (AML). Numerous studies have validated a diverse array of epigenetic therapies beyond DNA methylation inhibitors, ranging from histone deacetylase (HDAC) and demethylase inhibitors to non-coding RNA-based strategies [10]. A number of these drugs have received U.S. FDA approval, with several others currently in the developmental pipeline. Epigenetics offers a promising future for cancer therapeutics.

GSK-J4 serves as an inhibitor targeting KDM6 subfamily demethylases KDM6A and KDM6B, which catalyze the demethylation of H3K27me2/3. The therapeutic efficacy of GSK-J4 has been demonstrated in various tumor models. For instance, GSK-J4 effectively inhibits mantle cell lymphoma cell adhesion, survival, and drug resistance [18]. In retinoblastoma, GSK-J4 significantly suppresses cell growth and triggers apoptosis *in vitro* and *in vivo* [19]. Not limited to oncology, emerging evidence highlights the therapeutic potential of GSK-J4 in various inflammatory and autoimmune disorders, such as rheumatoid arthritis [20], diabetic nephropathy [21], Hashimoto’s thyroiditis [22], and intestinal inflammation [23]. However, the antitumor effect of GSK-J4 remains unexplored in the tumor-like lesion TSC.

In this study, we employ primary mouse embryonic fibroblasts (MEFs) with heterozygous deletion of *Tsc1* or *Tsc2* (*Tsc1^+/^^−^* and *Tsc2^+/^^−^* MEFs) to explore the effects of GSK-J4 on cell growth and its underlying mechanisms, unveiling a novel pathway: GSK-J4→p-PERK→c-Myc axis. Our findings suggest that GSK-J4 may offer a new therapeutic approach for TSC patients.

## 2. Results

### 2.1. Genetic Deficiencies in Either Tsc1 or Tsc2 Promote Proliferation and Inhibit Apoptosis

**Figure 1:**
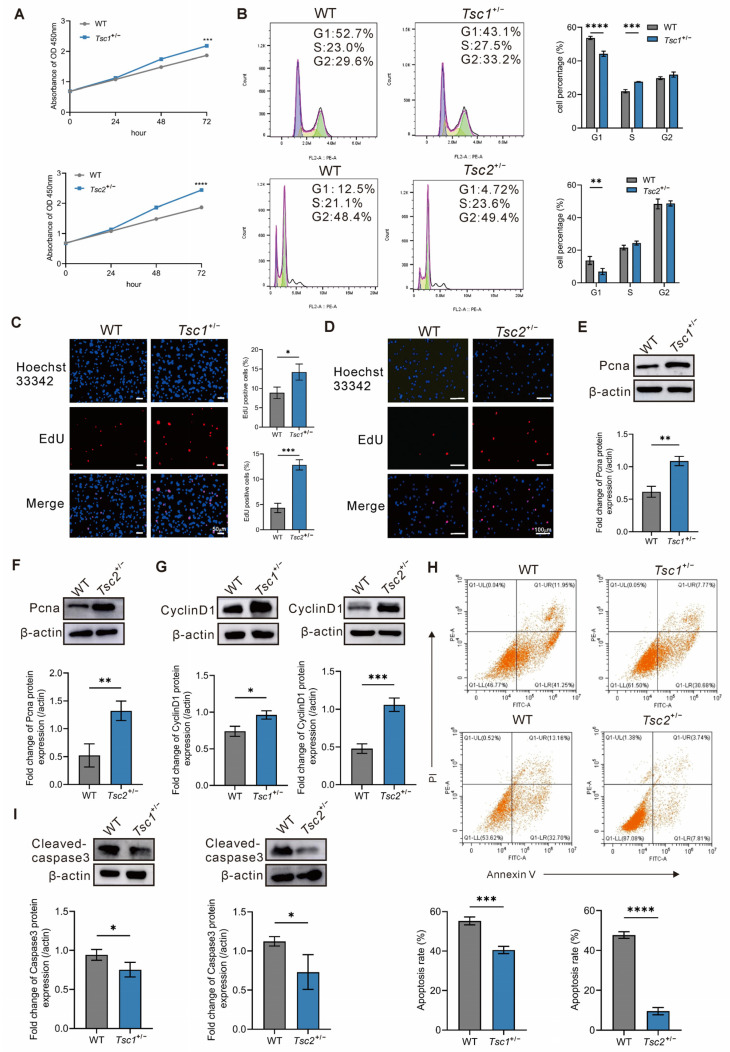
Analysis of cell proliferation and apoptosis in primary *Tsc1^+/−^* and *Tsc2^+/−^* MEFs. (**A**) Cell proliferation of primary *Tsc1^+/−^* and *Tsc2^+/−^* MEFs was assessed via CCK-8 assay. (**B**) Cell cycle distribution of primary *Tsc1^+/−^* and *Tsc2^+/−^* MEFs. Colors indicate G1 (blue), S (yellow), and G2 (green) phases. (**C**) EdU staining images of *Tsc1^+/−^* MEFs. Blue: Hoechst 33342−stained nuclei; Red: Edu−positive cells. Scale bar = 50 μm. (**D**) EdU staining images of *Tsc2^+/−^* MEFs. Blue: Hoechst 33342−stained nuclei; Red: Edu−positive cells. Scale bar = 100 μm. (**E**–**G**) Relative protein levels of Pcna and CyclinD1 in primary *Tsc1^+/−^* and *Tsc2^+/−^* MEFs were determined through Western blotting. (**H**) Cell apoptosis of primary *Tsc1^+/−^* and *Tsc2^+/−^* MEFs was analyzed by flow cytometry. (**I**) Relative protein levels of Cleaved-caspase3 in primary *Tsc1^+/−^* and *Tsc2^+/−^* MEFs were determined through Western blotting. * *p* < 0.05, ** *p* < 0.01, *** *p* < 0.001, **** *p* < 0.0001.

We firstly determined the levels of cell proliferation and apoptosis in primary *Tsc1^+/−^* and *Tsc2^+/−^* MEFs. As shown in Figure 1A, the CCK8 assays showed that the proliferation level of primary *Tsc1^+/−^* and *Tsc2^+/−^* MEFs was significantly increased. Meanwhile, flow cytometric analysis of the cell cycle revealed that loss of *Tsc1* or *Tsc2* expression introduced the low proportion of the G1 phase cells (Figure 1B). To further characterize the accelerated G1-S phase progression of cells with *Tsc1* or *Tsc2* defects, we performed EdU staining and Western blotting. EdU staining showed that the cell proliferation of primary *Tsc1^+/−^* and *Tsc2^+/−^* MEFs was significantly enhanced by quantitative and qualitative analyses (Figure 1C,D). Meanwhile, Western blotting analysis revealed that the deletion of *Tsc1* or *Tsc2* upregulated the proliferation markers Pcna and CyclinD1 (Figure 1E–G). Next, we quantified cell apoptosis using flow cytometry. It turned out that cell apoptosis decreased in primary *Tsc1^+/−^* and *Tsc2^+/−^* MEFs (Figure 1H). Moreover, Western blotting data showed the decreased expression of the apoptosis marker cleaved caspase-3 (Figure 1I). All results above demonstrate that *Tsc1* or *Tsc2* defects promote cell proliferation, accelerate the G1-S process, and suppress cell apoptosis.

### 2.2. Genetic Deficiencies in Either Tsc1 or Tsc2 Induce Lower Level of H3K27me3

**Figure 2:**
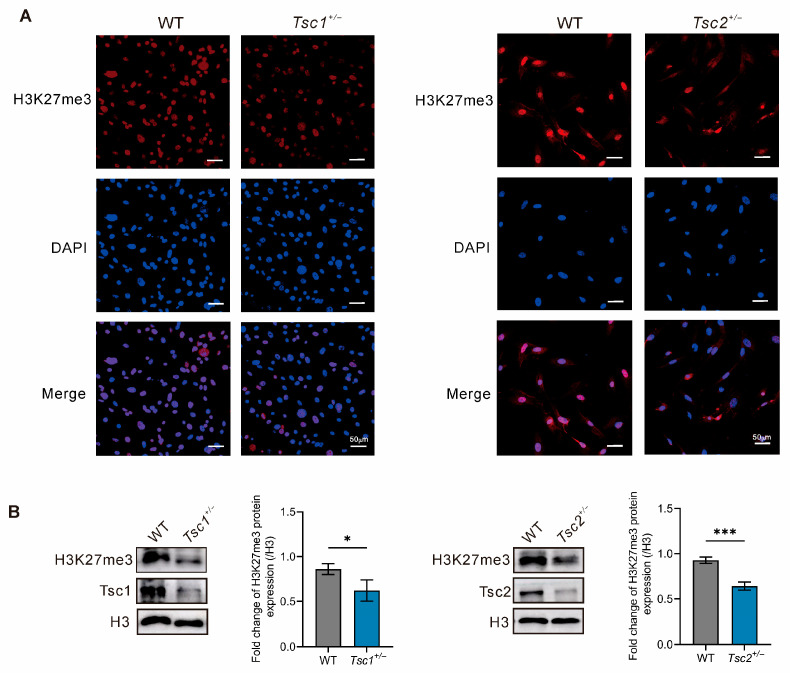
H3K27me3 levels in *Tsc1* or *Tsc2* deficient models. (**A**) Subcellular distribution of H3K27me3 in primary WT, *Tsc1^+/−^* and *Tsc2^+/−^* MEFs analyzed by immunofluorescence staining. Blue: DAPI-stained nuclei; Red: H3K27me3. Scale bars = 50 μm. (**B**) H3K27me3 protein expression levels in primary *Tsc1^+/−^* and *Tsc2^+/−^* MEFs were determined through Western blotting. * *p* < 0.05, *** *p* < 0.001.

H3K27me3 levels in genetic deficiencies of either *Tsc1* or *Tsc2* were analyzed by immunofluorescence and Western blotting. Immunofluorescence images revealed a weaker fluorescent intensity of the H3K27me3 signal in primary *Tsc1^+/−^* and *Tsc2^+/−^* MEFs in comparison to that in WT MEFs (Figure 2A). Meanwhile, Western blotting analysis demonstrated significantly reduced H3K27me3 in primary *Tsc1^+/−^* and *Tsc2^+/−^* MEFs compared with that in WT MEFs (Figure 2B). Those results above confirm that *Tsc1* or *Tsc2* deficiency leads to the low level of H3K27me3.

### 2.3. GSK-J4 Induces Cell Cycle Arrest and Apoptosis in Primary Tsc1^+/−^ and Tsc2^+/−^ MEFs

**Figure 3:**
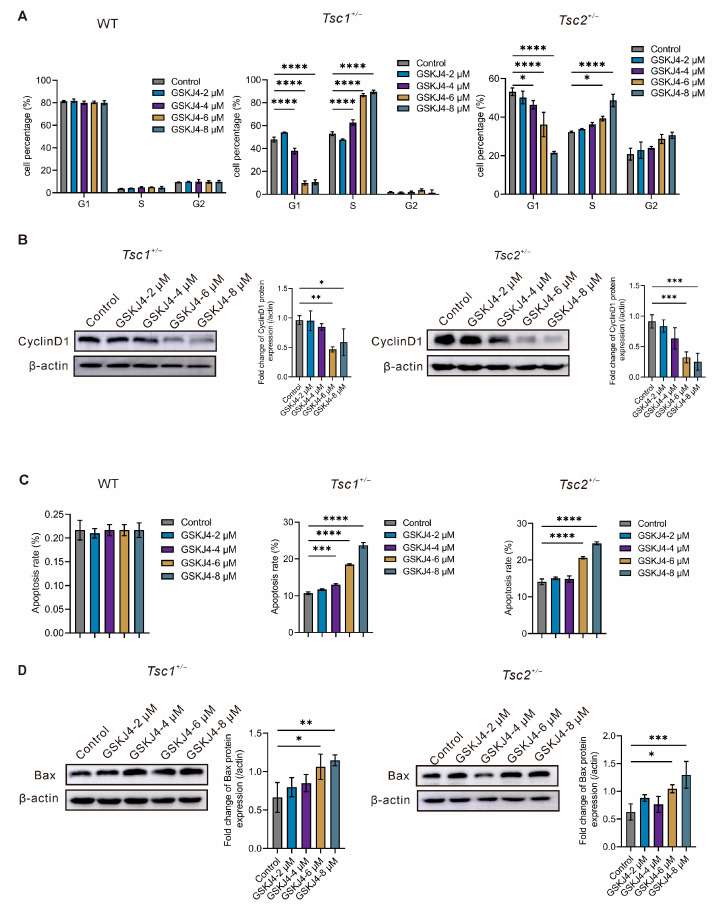
Effect of GSK-J4 treatment on cell cycle and apoptosis of primary MEFs at its different concentrations. (**A**) Quantitative analysis of the cell cycle distribution in primary MEFs (WT, *Tsc1^+/−^*, *Tsc2^+/−^*) treated with GSK-J4 at its different concentrations for 48 h. (**B**) CyclinD1 protein expression level was detected by Western blotting in primary *Tsc1^+/−^* and *Tsc2^+/−^* MEFs treated with GSK-J4 at its different concentrations for 48 h. (**C**) Quantitative analysis of apoptosis in primary WT, *Tsc1^+/−^*, and *Tsc2^+/−^* MEFs treated with varying concentrations of GSK-J4 for 48 h. (**D**) Bax protein expression level was detected by Western blotting in primary *Tsc1^+/−^* and *Tsc2^+/−^* MEFs treated with GSK-J4 at its different concentrations for 48 h. * *p* < 0.05, ** *p* < 0.01, *** *p* < 0.001, **** *p* < 0.0001.

We explored whether the low H3K27me3 levels were associated with proliferation and apoptosis in *Tsc1*- or *Tsc2*-deficient cells. GSK-J4, an inhibitor of the H3K27me3 demethylase, thereby elevates global H3K27me3 levels. Hence, we examined the influence of GSK-J4 on the cell cycle progression and apoptosis of primary MEFs using flow cytometry and Western blotting. The results showed that the administration of GSK-J4 led to a significant accumulation of cells in the S phase dose-dependently in primary *Tsc1^+/−^* and *Tsc2^+/−^* MEFs (Figure 3A and Supplementary Figure S1). Moreover, Western blotting revealed that CyclinD1 expression was significantly downregulated in primary *Tsc1^+/−^* and *Tsc2^+/−^* MEFs treated with GSK-J4 in a dose-dependent manner (Figure 3B). Surprisingly, GSK-J4 was ineffective for cell cycle distribution of primary WT-MEFs (Figure 3A and Supplementary Figure S1). Next, the roles of GSK-J4 in cell apoptosis of primary MEFs were investigated. The flow cytometric analysis demonstrated that GSK-J4 induced cell apoptosis in primary *Tsc1^+/−^* and *Tsc2^+/−^* MEFs dose-dependently (Figure 3C and Supplementary Figure S2). Moreover, the quantification of pro-apoptotic Bax expression by Western blotting showed that GSK-J4 treatment introduced an increase in Bax expression in primary *Tsc1^+/−^* and *Tsc2^+/−^* MEFs dose-dependently (Figure 3D). Surprisingly, GSK-J4 did not affect apoptosis of primary WT-MEFs (Figure 3C and Supplementary Figure S2). These findings suggest that GSK-J4 induces cell cycle arrest and apoptosis in primary *Tsc1^+/−^* and *Tsc2^+/−^* MEFs.

### 2.4. GSK-J4 Induces ER Stress in Primary Tsc1^+/−^ and Tsc2^+/−^ MEFs

**Figure 4:**
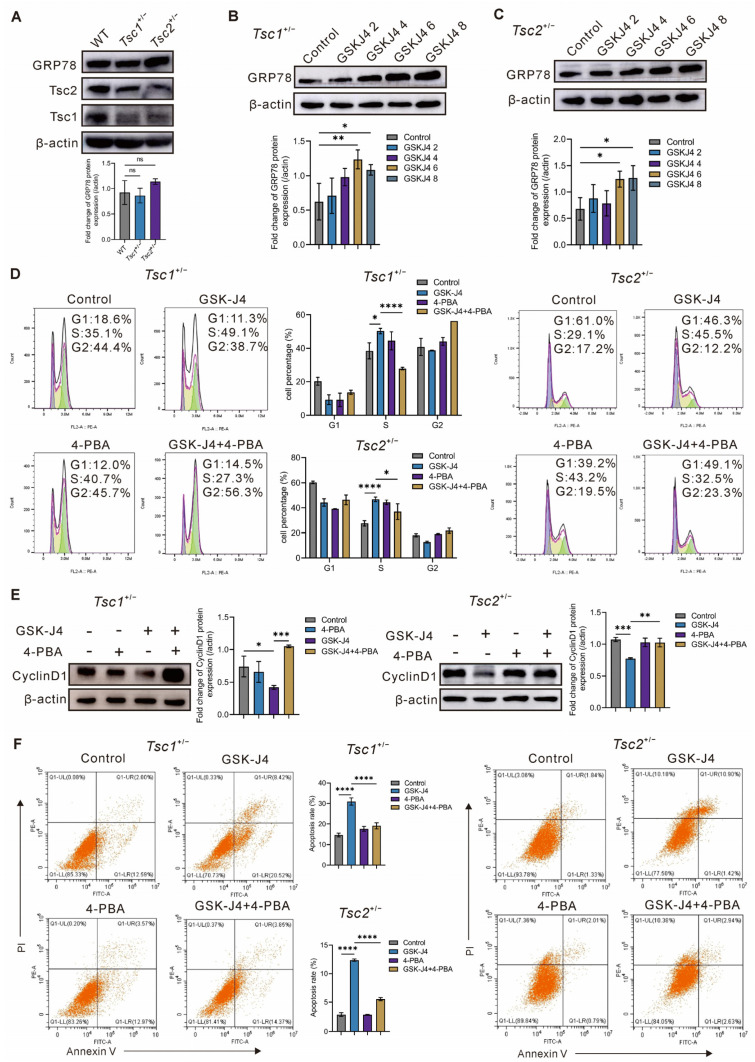
Induction of endoplasmic reticulum stress by GSK-J4 in primary *Tsc1^+/−^* and *Tsc2^+/−^* MEFs. (**A**) The basal GRP78 expression was analyzed between WT and *Tsc1-* or *Tsc2*-deficient primary MEFs. (**B**) The expression of GRP78 in *Tsc1^+/−^* MEFs treated with different concentrations of GSK-J4 was detected by Western blotting. (**C**) GRP78 expression in *Tsc2^+/−^* MEFs treated with the various concentrations of GSK-J4 was assessed by Western blotting. (**D**) Cell cycle distribution in *Tsc1^+/−^* and *Tsc2^+/−^* MEFs treated with 4-PBA combined with GSK-J4 (3 mM 4-PBA+ 6 μM GSK-J4). Colors indicate G1 (blue), S (yellow), and G2 (green) phases. (**E**) CyclinD1 expression levels in *Tsc1^+/−^* and *Tsc2^+/−^* MEFs treated with 4-PBA combined with GSK-J4 (3 mM 4-PBA+ 6 μM GSK-J4) were measured by Western blotting. (**F**) The proportion of apoptotic cells in *Tsc1^+/−^* and *Tsc2^+/−^* MEFs treated with 4-PBA combined with GSK-J4 (3 mM 4-PBA+ 6 μM GSK-J4). ns, not significant, * *p* < 0.05, ** *p* < 0.01, *** *p* < 0.001, **** *p* < 0.0001.

Endoplasmic reticulum stress (ERS) has been reported to affect cellular proliferation and apoptosis. GRP78 is an ERS sensor that serves a critical role in the rescue mechanism of the unfolded protein response (UPR). To investigate whether GSK-J4 affected proliferation and apoptosis in *Tsc1*- or *Tsc2*-deficient cells through ERS activation, GRP78 expression was measured by Western blotting. First, we assessed primary MEF-based GRP78 expression levels. The data indicated no significant difference in GRP78 levels among primary WT, *Tsc1^+/−^*, and *Tsc2^+/−^* MEFs (Figure 4A). Next, we examined GRP78 expression levels in MEFs exposed to a range of GSK-J4 concentrations. The results showed that GRP78 expression remained unaltered in WT cells but showed a progressive upregulation in *Tsc1*- or *Tsc2*-deficient cells as GSK-J4 concentration increased (Figure 4B,C and Supplementary Figure S3A). Collectively, these findings indicate that GSK-J4 activates ERS in *Tsc1*- or *Tsc2*-deficient cells.

To further clarify the role of GSK-J4 in modulating proliferation and apoptosis of primary *Tsc1^+/−^* and *Tsc2^+/−^* MEFs through ERS activation, we detected the cell cycle progression and apoptosis after co-treatment of GSK-J4 with 4-phenylbutyric acid (4-PBA, the ERS inhibitor). According to Figure 4D, the accumulation of S phase cells was decreased more significantly with combinatorial treatment of GSK-J4 with 4-PBA than that in the GSK-J4 monotherapy group. The Western blotting data revealed that co-treatment of GSK-J4 with 4-PBA upregulated the CyclinD1 expression compared to that in the GSK-J4 monotherapy group in *Tsc1-* or *Tsc2*-deficient cells (Figure 4E). Meanwhile, our investigation by flow cytometry indicated that the co-treatment of GSK-J4 with 4-PBA reduced the apoptosis of primary *Tsc1^+/−^* and *Tsc2^+/−^* MEFs compared to that in the GSK-J4-alone treated cells (Figure 4F). Moreover, we found that 4-PBA administration had no significant influence on cell cycle progression and apoptosis in primary WT-MEFs treated with GSK-J4 (Supplementary Figure S3B,C), thereby demonstrating that GSK-J4 exerts anti-proliferative and pro-apoptotic effects on *Tsc1-* or *Tsc2*-deficient cells through ERS activation.

### 2.5. GSK-J4 Activates the PERK Pathway to Inhibit c-Myc Expression

**Figure 5:**
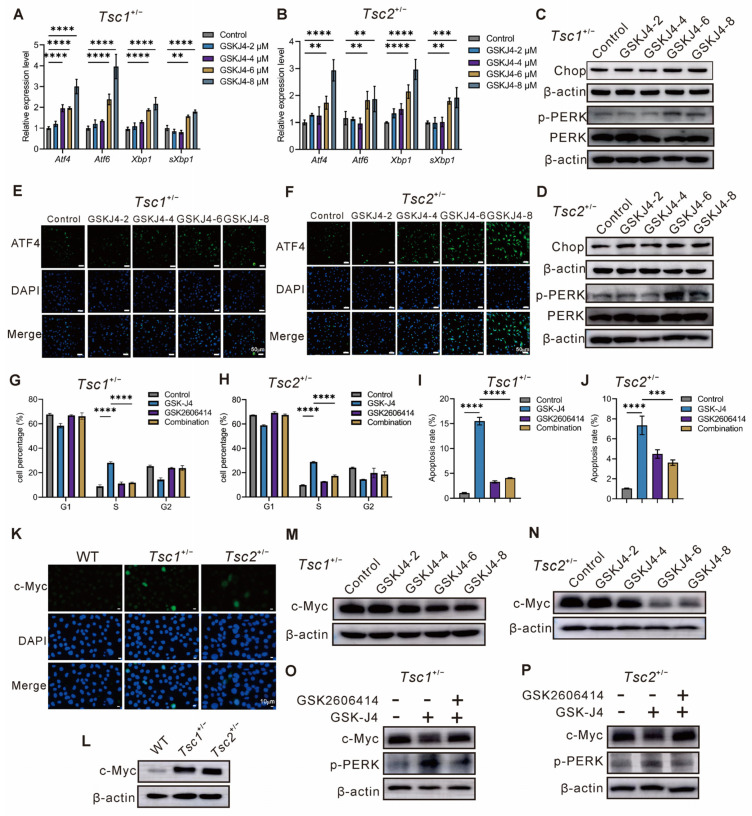
Inhibition of c-Myc expression by GSK-J4 through PERK activation. (**A**,**B**) Transcription factors involved in endoplasmic reticulum stress detected by qPCR in *Tsc1^+/−^* and *Tsc2^+/−^* MEFs treated with different concentrations of GSK-J4. (**C**,**D**) p-PERK and Chop protein expression levels in *Tsc1^+/−^* and *Tsc2^+/−^* MEFs treated with different concentrations of GSK-J4 detected by Western blotting. (**E**,**F**) Subcellular distribution of ATF4 in *Tsc1^+/−^* and *Tsc2^+/−^* MEFs treated with different concentrations of GSK-J4 analyzed by immunofluorescence staining. Blue: DAPI−stained nuclei; Green: ATF4. Scale bars = 50 μm. (**G**,**H**) Quantitative analysis of the cell cycle distribution in each group after drug treatment for *Tsc1^+/−^* and *Tsc2^+/−^* MEFs [Control, GSK-J4 (6 μM), GSK2606414 (10 μM), GSK-J4 (6 μM) + GSK2606414 (10 μM)]. (**I**,**J**) Quantitative analysis of apoptosis in each group after drug treatment for *Tsc1^+/−^* and *Tsc2^+/−^* MEFs [Control, GSK-J4 (6 μM), GSK2606414 (10 μM), GSK-J4 (6 μM) + GSK2606414 (10 μM)]. (**K**) Subcellular distribution of c-Myc in primary WT, *Tsc1^+/−^*, and *Tsc2^+/−^* MEFs analyzed by immunofluorescence staining. Blue: DAPI−stained nuclei; Green: c-Myc. Scale bars = 10 μm. (**L**) c-Myc protein expression levels in primary WT, *Tsc1^+/−^*, and *Tsc2^+/−^* MEFs detected by Western blotting. (**M**,**N**) c-Myc protein expression levels in *Tsc1^+/−^* and *Tsc2^+/−^* MEFs treated with different concentrations of GSK-J4 detected by Western blotting. (**O**,**P**) c-Myc protein expression levels in each group after drug treatment for *Tsc1^+/−^* MEFs and *Tsc2^+/−^* MEFs [Control, GSK-J4 (6 μM), GSK-J4 (6 μM) + GSK2606414 (10 μM)] detected by Western blotting. ** *p* < 0.01, *** *p* < 0.001, **** *p* < 0.0001.

ERS is mainly regulated through the UPR pathway, which is centered on three transmembrane sensor signaling pathways: IRE1, PERK, and ATF6, and synergistically regulates protein folding, degradation, or cell-fate decisions. In this study, qPCR analysis demonstrated that the mRNA levels of the pathway-specific transcription factors *Xbp1*, *sXbp1*, *Atf4*, and *Atf6* were upregulated dose-dependently by GSK-J4 in both *Tsc1-* or *Tsc2*-deficient MEFs (Figure 5A,B), indicating that GSK-J4 comprehensively triggered the activation of three ER stress pathways. The PERK-ATF4 axis is a central compartment of the UPR that decisively controls cell fate by mediating the transition from adaptive survival to apoptosis. Moreover, CHOP, serving as a key pro-apoptotic mediator, is centrally regulated by the PERK signaling axis. Therefore, we proposed that GSK-J4 activated the PERK-ATF4 axis, thereby upregulating CHOP and ultimately resulting in the inhibition of proliferation and the induction of apoptosis in primary *Tsc1^+/−^* and *Tsc2^+/−^* MEFs. We then examined the protein levels of p-PERK and Chop. Western blotting analysis showed that both p-PERK and Chop levels were not obviously altered at low concentrations but significantly increased at high concentrations in a concentration-dependent manner after GSK-J4 treatment (Figure 5C,D). Moreover, immunofluorescence staining data showed that ATF4 level was dose-dependently upregulated after GSK-J4 treatment in *Tsc1^+/−^* and *Tsc2^+/−^* MEFs (Figure 5E,F). Following subcellular fractionation for the isolation of cytoplasmic and nuclear compartments, the data indicated that the localization of ATF4 was intra-nuclear, with significantly increased expression levels following GSK-J4 treatment in *Tsc1-* or *Tsc2*-deficient cells (Supplementary Figure S4A). These data showed that GSK-J4 comprehensively activated three ER stress pathways, thereby engaging the core PERK-ATF4-CHOP axis. To investigate whether PERK activation mediated the effects of GSK-J4 on proliferation and apoptosis in *Tsc1-* or *Tsc2*-deficient cells, we employed the PERK inhibitor GSK2606414. The combined treatment with GSK-J4 and the PERK inhibitor attenuated S-phase arrest and apoptosis compared to GSK-J4 treatment alone (Figure 5G–J and Supplementary Figure S4B,C), suggesting that GSK-J4 regulated proliferation and apoptosis in *Tsc1-* or *Tsc2*-deficient cells through activation of the ERS-dependent PERK pathway.

PERK activation effectively suppresses global protein translation through phosphorylation of substrate eIF2α [24]. Meanwhile, dysregulated c-Myc functions as a key pathogenic driver in human tumor development. Therefore, to determine whether PERK-mediated signaling regulates c-Myc expression or activity, we directly investigate potential functional interactions between them. Initially, we assessed c-Myc expression following *Tsc1* or *Tsc2* deficiency. Immunofluorescence images revealed a stronger fluorescent intensity of c-Myc signal in primary *Tsc1^+/−^* and *Tsc2^+/−^* MEFs in comparison to that in WT MEFs (Figure 5K). Meanwhile, Western blotting analysis demonstrated that c-Myc was generally upregulated in *Tsc1-* or *Tsc2*-deficient models (Figure 5L). A similar trend was also observed in qPCR analysis (Supplementary Figure S4D). Subsequently, we examined c-Myc levels in *Tsc1-* or *Tsc2*-deficient cells upon exposure to a dose range of GSK-J4. Western blotting analysis revealed that c-Myc protein levels were reduced after GSK-J4 treatment in both *Tsc1-* or *Tsc2*-deficient cells in a dose-dependent manner (Figure 5M,N). Consistently, qPCR analysis demonstrated that GSK-J4 treatment reduced *c-Myc* mRNA levels (Supplementary Figure S4E). Given that GSK-J4 activated the PERK pathway, we hypothesized that the downregulation of c-Myc by GSK-J4 was mediated through PERK activation. Consistent with this, treatment with a PERK inhibitor reversed the GSK-J4-induced decrease in c-Myc protein in *Tsc1-* or *Tsc2*-deficient cells (Figure 5O,P). Taken together, GSK-J4 activates the PERK signaling pathway, which in turn leads to the suppression of c-Myc expression.

### 2.6. GSK-J4 Synergizes with Rapamycin to Ameliorate Tsc-Deficiency Phenotypes

**Figure 6:**
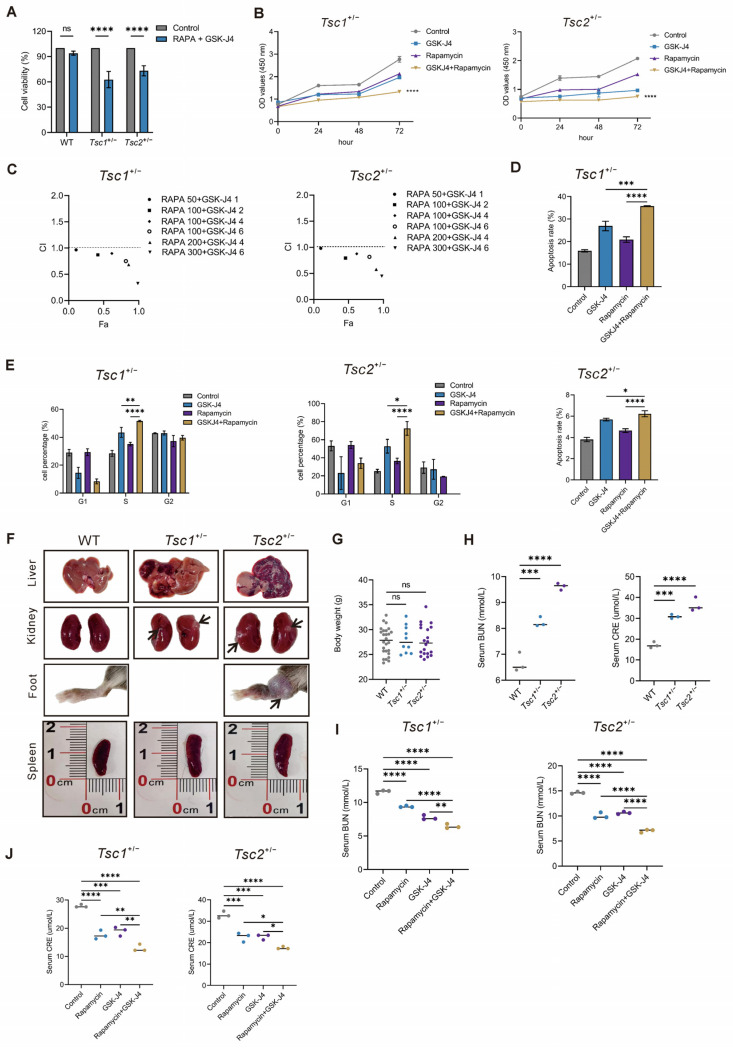
Synergistic growth suppression and reduced kidney injury by GSK-J4 and rapamycin. (**A**) Cell proliferation in primary WT, *Tsc1^+/−^*, and *Tsc2^+/−^* MEFs treated with rapamycin (100 nM) combined with GSK-J4 (2 μM) was assessed via CCK-8 assay. (**B**) Cell proliferation in cells treated with the GSK-J4 (2 μM) and rapamycin (100 nM), individually and in combination, was tested by CCK-8 assay. (**C**) The combined index (CI) of rapamycin and GSK-J4 was analyzed by CompuSyn software (version 1.0; ComboSyn, Inc., Paramus, NJ, USA). (**D**) Quantitative analysis of apoptosis in primary *Tsc1^+/−^* and *Tsc2^+/−^* MEFs treated with the GSK- J4 (2 μM) and rapamycin (100 nM), individually and in combination. (**E**) Quantitative analysis of the cell cycle distribution in cells (primary *Tsc1^+/−^* and *Tsc2^+/−^* MEFs) treated with the GSK-J4 (2 μM) and rapamycin (100 nM), individually and in combination. (**F**) Gross morphological analysis of tissues in 12- to 13-month-old mice (WT, *Tsc1^+/−^*, *Tsc2^+/−^*). (**G**) Mouse body weight was examined (WT, *Tsc1^+/−^*, and *Tsc2^+/−^*). (**H**) The CRE and BUN levels detected (WT, *Tsc1^+/−^*, and *Tsc2^+/−^*), *n* = 3 for each genotype. (**I**,**J**) Twelve- to thirteen-month-old male *Tsc1^+/−^* and *Tsc2^+/−^* C57BL/6 mice (*n* = 3 per group) were treated with control, GSK-J4 (0.4 mg/kg, s.c. daily for 10 days), rapamycin (1 mg/kg, i.p. every other day for 20 days), or their combination. The BUN and CRE levels were measured. All animal data are presented as individual data points (each dot represents one mouse) with horizontal lines indicating the mean value of each group. Data are presented as mean ± SD. ns, not significant, * *p* < 0.05, ** *p* < 0.01, *** *p* < 0.001, **** *p* < 0.0001 (one-way ANOVA).

Rapamycin, an mTORC1 inhibitor, can be used as a first-line agent in TSC therapy. We next aimed to clarify the role of the GSK-J4 combination with rapamycin in the TSC treatment. First, we investigated the effect of the combined treatment on the proliferation of primary *Tsc1^+/−^* and *Tsc2^+/−^* MEFs at 48 h after treatment. The results showed that the combination of GSK-J4 and rapamycin exerted no significant effect on primary WT-MEFs but significantly inhibited the proliferation of *Tsc1-* or *Tsc2*-defective MEFs compared with the control group (Figure 6A). To evaluate the potential synergistic efficacy of the drug combination over single drug treatment, the primary *Tsc1^+/−^* and *Tsc2^+/−^* MEFs were exposed to 100 nM rapamycin, 2 μM GSK-J4, and 100 nM rapamycin + 2 μM GSK-J4 post 48 h. As shown in Figure 6B, the CCK-8 assays showed that cell viability of primary *Tsc1^+/−^* and *Tsc2^+/−^* MEFs was much more significantly decreased with the combinatorial treatment of rapamycin and GSK-J4 in comparison to that of single-drug treatment with either rapamycin or GSK-J4. CompuSyn analysis confirmed a combination index (CI) of less than 1 for rapamycin and GSK-J4, indicating synergistic inhibition of proliferation in primary *Tsc1-* or *Tsc2*-defective MEFs (Figure 6C).

We hypothesized that this synergistic anti-proliferative effect was mediated by both the extension of S phase and activation of apoptosis. To test this, we assessed apoptosis induction in primary *Tsc1-* or *Tsc2*-deficient MEFs using flow cytometry. As we hypothesized, the drug combination resulted in a significantly higher percentage of apoptotic cells than either drug alone (Figure 6D and Supplementary Figure S5B), demonstrating that the enhanced apoptosis may contribute to the synergistic inhibition of *Tsc1^+/−^* and *Tsc2^+/−^* MEFs growth. Rapamycin has been shown to cause cell cycle arrest. Our cell cycle assays showed that GSK-J4 triggered a G1/S phase arrest in primary *Tsc1^+/−^* and *Tsc2^+/−^* MEFs. Next, we investigated the cell cycle progression of primary *Tsc1^+/−^* and *Tsc2^+/−^* MEFs under the condition of combinatorial treatment of the two drugs above. The results demonstrated that the combinatorial treatment significantly induced G1/S phase arrest and prolonged S phase progression in primary *Tsc1^+/−^* and *Tsc2^+/−^* MEFs (Figure 6E and Supplementary Figure S5A). We also demonstrated that the knockdown of *Kdm6a* increased the sensitivity of *Tsc1^−/^^−^* and *Tsc2^−/^^−^* MEFs to rapamycin in cell viability and cell apoptosis (Supplementary Figure S6A,B), further supporting that rapamycin and GSK-J4 had synergistic effects on both anti-proliferation and pro-apoptosis in primary *Tsc1^+/−^* and *Tsc2^+/−^* MEFs.

We investigated the therapeutic potential of GSK-J4 in TSC mouse models. Initial phenotypic characterization confirmed that while *Tsc1* or *Tsc2* deficiency did not significantly alter mouse body weight, it induced a range of pathological features across multiple organs, such as tumor formation, abnormal vascularization, and diffuse parenchymal hyperplasia (Figure 6F,G and Supplementary Figure S7). These models also displayed impaired renal function, marked by significantly higher blood urea nitrogen (BUN) and creatinine (CRE) levels than controls (Figure 6H), indicating that the heterozygous knockout models recapitulated the multi-organ involvement observed in TSC patients.

Subsequently, we assessed the efficacy of the mTORC1 inhibitor (using rapamycin) and GSK-J4 *in vivo*. Either GSK-J4 or rapamycin monotherapy markedly reduced serum BUN and CRE levels, with the combination of both agents producing the most substantial reduction (Figure 6I,J). Collectively, these results demonstrate that GSK-J4 represents a promising combinatorial therapeutic partner for rapamycin in the treatment of TSC.

## 3. Discussion

Defects in *TSC1* or *TSC2* disrupt TSC formation, leading to mTORC1 hyperactivation. This mechanism has enabled the development of targeted therapies for TSC. mTORC1 inhibitor therapies have greatly improved the prognosis of TSC patients by effectively controlling tumor growth and alleviating associated neurological and psychiatric symptoms [2]. However, TSC patients exhibit significant clinical variability, including the heterogeneity of tubers and suboptimal tumor control with existing therapies. For example, tumor recurrence is commonly observed following the withdrawal of sirolimus [25]. Meanwhile, the administration of mTORC1 inhibitors carries a risk of adverse effects, including oral ulcers, hyperlipidemia, and hyperglycemia, leading to poor patient compliance. Thus, developing novel therapeutic approaches or optimizing combination strategies is crucial for more effective and less toxic TSC therapies. In this study, we successfully validated *Tsc1/Tsc2*-defective mouse models as a tool for studying TSC pathogenesis, based on the highly consistent with the clinical presentation of TSC patients in both histomorphological and biochemical profiles. Moreover, we isolated primary MEFs from *Tsc1^+/−^* and *Tsc2^+/−^* mice as cellular models, which displayed elevated proliferation and reduced apoptosis, consistent with the predominant tumor growth observed in TSC patients (Figure 1).

Some studies suggest that the mTORC1 pathway and the *TSC* gene are regulated by epigenetic mechanisms. Accumulating evidence indicates that such epigenetic modifications may contribute to gene expression regulation and are associated with the clinical heterogeneity observed in TSC [26]. For instance, methylation of the *TSC2* promoter reduces TSC2 protein expression, thereby promoting the development of TSC-associated angiomyolipomas [27]. Epigenetic alterations have also been implicated in the inflammatory aspects of TSC. Notably, significant hypomethylation of the interleukin-1β (IL-1β) promoter has been identified in TSC brain tissue, particularly within and surrounding tuber nodules, and this hypomethylation correlates positively with increased IL-1β expression, representing a characteristic feature of TSC neuropathology [28]. In addition, reduced histone H3 acetylation levels have been observed in the hippocampus of *Tsc2* heterozygous knockout mice, associated with impaired synaptic plasticity and epileptic phenotypes. Interestingly, these phenotypes occur even in the absence of significant alterations in mTORC1 signaling, suggesting that neurophysiological symptoms may be mediated by non-mTORC1-dependent epigenetic modifications [29]. Furthermore, microRNAs influence TSC pathogenesis through the regulation of gene expression, mTORC1 signaling, and cellular differentiation [30]. However, the role of histone methylation in TSC pathogenesis remains unreported.

Dysregulation of H3K27me3 contributes to multiple pathological processes, including cancer. No study has defined if H3K27me3 levels change during TSC pathogenesis. In this study, we observed that H3K27me3 levels decreased in primary *Tsc1^+/−^* and *Tsc2^+/−^* MEFs (Figure 2). The change in H3K27me3 differs from some previous reports using *p53*-knockout immortalized MEF cell lines [31]. This difference may be attributed to the distinct cellular models: our study is performed in primary MEFs that maintain a relatively normal physiological state, while the cell lines may lead to altered epigenetic regulation. Since H3K27me3 is highly context-dependent and tightly regulated by p53 signaling, these divergent results are biologically reasonable and complementary rather than contradictory. GSK-J4, an inhibitor of KDM6A/B, increases H3K27me3 levels and suppresses aberrant growth both *in vitro* and *in vivo* across multiple cancers (e.g., neuroblastoma, glioma, and breast cancer) [32,33,34]. Nevertheless, GSK-J4 remains confined to preclinical studies. Our research confirmed that GSK-J4 specifically inhibited cell cycle progression and activated apoptosis of primary *Tsc1^+/−^* and *Tsc2^+/−^* MEFs dose-dependently, but it had no significant effects on the proliferation and apoptosis of WT MEFs (Figure 3). This suggested that the effect of GSK-J4 may be contingent upon global H3K27me levels.

ERS is a cellular stress response triggered by the aggregation of unfolded or misfolded proteins within the endoplasmic reticulum. When the demand for protein folding exceeds the processing capacity in the endoplasmic reticulum, the unfolded protein response is activated, which restores homeostasis. Moderate ERS helps cells to adapt to stress, but sustained or intense ERS perturbs numerous cellular processes, including growth, cell cycle progression, and apoptosis. In our study, GSK-J4 activated ERS in *Tsc1*- or *Tsc2*-deficient cells. Meanwhile, we detected no significant difference in ER stress levels among WT, *Tsc1*-deficient, and *Tsc2*-deficient cells, indicating that ER stress induction appeared to be mediated exclusively by GSK-J4 and was independent of the underlying genotype in *Tsc*-deficient models. Moreover, we confirmed that GSK-J4 induced cell cycle arrest and apoptosis through ERS in *Tsc1*- or *Tsc2*-deficient cells using the ER stress inhibitor 4-PBA (Figure 4). However, this effect was not observed on primary WT MEFs, consistent with our observation that GSK-J4 treatment did not induce ER stress in WT cells.

ERS activates three signaling pathways, orchestrated by the sensors PERK, IRE1α, and ATF6, which collectively regulate cell cycle progression and apoptosis [35]. As a key pathway for ERS-induced apoptosis, the PERK-ATF4-CHOP axis is initiated by PERK, an ER transmembrane protein that is a major sensor of the UPR. Upon PERK activation, it phosphorylates eIF2α, leading to the regulation of cellular autophagy and apoptosis. The PERK/eIF2α axis upregulation underlies the 17-AAG-induced apoptosis in breast cancer cells [36]. The paraquat-induced apoptosis in human lung epithelioid cells (A549 cells) is elevated, and GRP78, p-PERK, and p-eIF2α levels are upregulated, which proves that the PERK/p-eIF2α pathway is involved in the process of apoptosis [37]. However, another study has found that *PERK* knockdown may exacerbate ER stress in osteosarcoma cells, thereby promoting cell apoptosis [38]. These studies suggest that the function of PERK in cancer is highly context-dependent. In this study, we observed that GSK-J4 treatment upregulated the expression of p-PERK, Chop, and ATF4 and increased intra-nuclear ATF4 in a dose-dependent manner by immunofluorescence staining (Figure 5). In summary, GSK-J4-induced ER imbalance triggered the UPR, as evidenced by the activation of both ATF4 and PERK signaling pathways. Ultimately, Chop functioned as the downstream apoptotic effector, executing programmed cell death through Bax upregulation in TSC tumor cells.

PERK kinase activation has been described through two distinct mechanisms. In the classical model, the accumulation of unfolded or misfolded proteins during ERS triggers the dissociation of GRP78 from PERK, leading to PERK activation [39]. In the non-classical model, PERK is directly bound and activated by STING via its cytosolic domain [40]. This study demonstrated that GSK-J4 activated PERK in an ER stress-dependent manner, thereby suppressing c-Myc protein expression. As a global transcription factor, c-Myc regulates approximately 15% of human genes, which are critically involved in cell cycle progression, metabolic pathways, ribosome biogenesis, and protein synthesis [41]. Therefore, TSC tumors with c-Myc activation would exhibit excessive protein synthesis of c-Myc itself and its downstream targets, making them susceptible to significant suppression by the GSK-J4-PERK axis.

GSK-J4 has been established as an effective chemosensitizer that demonstrates synergistic potential when combined with various antineoplastic agents. Recently, the study has shown that GSK-J4 co-treated with cisplatin can dramatically promote the inhibition and regression of testicular germ cell tumors (TGCT) *in vivo* [42]. In high-risk neuroblastoma, the WIP1 inhibitor and GSK-J4 exhibit synergistic anti-tumor activity through enhanced activation of p53-mediated signaling [34]. GSK-J4 enhances the chemosensitivity of diffuse large B-cell lymphoma to both doxorubicin and bortezomib [43]. Moreover, GSK-J4 and donafenib exhibit a synthetic lethality relationship, effectively killing liver cancer cells [44]. Rapamycin is approved for TSC therapy as an inhibitor of mTORC1. Our results demonstrated that the combination of GSK-J4 and rapamycin had synergism on the inhibition of cell growth in primary *Tsc1^+/−^* and *Tsc2^+/−^* MEFs compared to either treatment alone. These results demonstrate the mechanism wherein H3K27me3 levels modulate the anti-proliferative efficacy of mTOR inhibitors, which is in agreement with a previous study showing that GSK126 (an EZH2 inhibitor that reduces H3K27me3) attenuates the growth-inhibitory effects of INK128 in MCF-7 cells [31]. Meanwhile, both GSK-J4 and rapamycin alleviated renal injury caused by *Tsc1* or *Tsc2* deficiency, with their combination exhibiting a markedly enhanced therapeutic effect *in vivo*, indicating the potential for precision medicine in TSC (Figure 6). It will be interesting to investigate whether the combination of GSK-J4 and rapamycin exerts its synergistic effect via modulating H3K27me3 in TSC models, which will be explored in our future study.

Beyond elucidating its therapeutic efficacy, evaluating the pharmacokinetics and toxicity profile of GSK-J4 is essential to facilitate its clinical translation. Although previous studies have explored alternative therapeutic indications for GSK-J4, all available preclinical data remain confined to cellular and animal model studies. Notably, in contrast to the therapeutic efficacy observed in disease models, GSK-J4 does not induce apparent toxicity across multiple independent studies [45,46]. Consistent with these *in vivo* findings, *in vitro* assays have demonstrated that treatment with GSK-J4 induces cell cycle arrest and cell death in different kinds of cancer cells with dismal toxicity to normal cells [47,48,49]. Collectively, these results support a favorable safety profile for GSK-J4 at the doses tested. Moreover, while definitive pharmacokinetic parameters of GSK-J4 have not yet been reported, existing evidence suggests that it possesses favorable properties, such as highly efficient cell permeability [50]. This property facilitates its access to the brain and kidneys, the major organs affected by TSC, making it a promising candidate for TSC therapies. While further preclinical studies are required to fully characterize its long-term safety profile and optimal dosing regimen, the current findings underscore the considerable translational potential of GSK-J4 for the treatment of TSC.

While these preliminary results are promising, further investigation through expanded preclinical studies and clinical trials is warranted. Notably, therapeutic strategies targeting H3K27me3 modification (e.g., Polycomb Repressive Complex 2 (PRC2) inhibitors) have already advanced to clinical trials, demonstrating their translational potentials in the clinic. Based on our findings, GSK-J4 represents a promising candidate for future TSC therapy development.

## 4. Materials and Methods

### 4.1. Cell Cultures

The MEF lines with *Tsc1^−/−^* and *Tsc2^−/−^* were acquired from the laboratory of Professor Hongbing Zhang at Peking Union Medical College. The MEFs, both primary isolates and established lines, were grown in high-glucose Dulbecco’s Modified Eagle Medium (DMEM, BOSTER, Wuhan, China), which was supplemented with 10% heat-inactivated fetal bovine serum (FBS, SORFA, Huzhou, China) under standard culture conditions (37 °C, 5% CO_2_ in a humidified atmosphere).

### 4.2. Euthanasia of Mice by Cervical Dislocation

Ethical approval for this research was granted by the Committee of Scientific Research at Shanxi University (CSRSX) under approval number SXULL2020032. All animal procedures were performed in compliance with the NIH Guide for the Care and Use of Laboratory Animals and reported following the ARRIVE 2.0 guidelines.

The mice were euthanized by cervical dislocation under 4–5% isoflurane anesthesia. Briefly, the mouse was gently restrained by one hand, and rapid, firm pressure was applied to the base of the skull while pulling the tail caudally with constant force to dislocate the cervical vertebrae and induce sudden death.

### 4.3. Isolation of Primary MEFs

Male *Tsc1* or *Tsc2* heterozygous knockout mice were crossed with wild-type female mice. Timed-pregnant mice (E12.5) were euthanized, and the embryos were harvested. Embryos were aseptically isolated in cold Dulbecco’s phosphate-buffered saline (DPBS). Embryonic heads and visceral organs were surgically removed. Remaining trunks were minced into 1 mm^3^ fragments using sterile scalpel blades. Tissue fragments were digested in 0.05% trypsin-ethylenediaminetetraacetic acid (EDTA, *w*/*v*). Enzymatic tissue digestion proceeded for 20–30 min at 37 °C with intermittent gentle agitation. An equal volume of DMEM supplemented with 10% FBS was added to neutralize trypsin activity. The cell suspensions were filtered through 200-mesh nylon membranes (Solarbio, Beijing, China). Filtered suspensions were centrifuged at 4 °C (300× *g*, 5 min). Following resuspension in complete DMEM, the cell pellets were plated into six-well plates, and the plates were placed back into the standard incubator conditions until 90% confluence of cells before passaging. Taking advantage of the strong adhesive properties of fibroblasts, primary mouse embryonic fibroblasts (MEFs) were expanded through three passages to establish a pure population. These MEFs at passages 4–8 (P4–P8) were genotyped and used for the subsequent experiments.

### 4.4. CCK-8 Assay

Cell viability was determined using the Cell Counting Kit-8 (CCK-8, NCM Biotech, Suzhou, China) as directed. For this assay, a seeding density of 1 × 10^4^ cells per well in 96-well plates was used. Following cell adhesion, experimental treatments were initiated according to the designated protocol. After treatment, each well was supplemented with CCK-8 reagent (10 μL). Following a 2 h incubation at 37 °C, the absorbance (OD) of cells at a wavelength of 450 nm was measured in each well.

### 4.5. EdU Incorporation Assay

Cells were seeded in 12-well plates (2 × 10^4^ cells/well) and subsequently maintained in culture for 24 h. To detect newly synthesized DNA, we used the BeyoClick™ EdU-555 Cell Proliferation Kit (C0075S, Beyotime Biotech Inc, Shanghai, China) according to the provided instructions. EdU-positive cells were quantified via fluorescence microscopy. The evaluation of digital images was performed using ImageJ software (version 1.53t; National Institutes of Health, Bethesda, MD, USA).

### 4.6. Cell Cycle Analysis

We performed cell cycle analysis by following the protocol provided with the DNA Quantitation Assay kit (Solarbio, Beijing, China). After being washed twice with ice-cold phosphate-buffered saline (PBS, pH 7.4), cells were precipitated by centrifugation (1500 rpm, 5 min, 4 °C), and then the cell pellets were subjected to overnight incubation at 4 °C in 500 μL of ice-cold 70% ethanol (in PBS). Cells underwent washes twice to eliminate residual ethanol. Following resuspension in 100 μL RNase A solution (37 °C, 30 min), cells were incubated at 4 °C in the dark for 30 min with the addition of 400 μL propidium iodide (PI) staining solution; finally, cells were detected using flow cytometry.

### 4.7. Apoptosis Assay

The cell apoptosis was quantified using the Annexin V-AbFluor™ 488/Apoptosis Detection kit (Abbkine, Wuhan, China). Cells were washed with pre-cooled PBS (pH 7.4), subsequently harvested by trypsinization (0.25% trypsin, without EDTA), and finally centrifuged at 300× *g* for 5 min at 4 °C. For subsequent analysis, approximately 2 × 10^5^ cells were prepared. Cells were resuspended with 100 μL of 1× Annexin V Binding Buffer containing Annexin V-AbFluor™ 488 (5 μL) and PI (2 μL). The negative control received buffer-only treatment (no Annexin V or PI added); cells were incubated (15 min, room temperature) under light-protected conditions, and then added 1× Annexin V Binding Buffer (400 μL). Cell suspensions were subjected to flow cytometric analysis within 0.5 h after staining.

### 4.8. RNA Extraction and Real-Time Quantitative PCR (RT-qPCR)

Total RNA was extracted with TRIzol reagent (Invitrogen, Carlsbad, CA, USA) according to the recommended protocol. cDNA synthesis was performed with Prime Script RT Master Mix (TAKARA, Japan), and qPCR reactions were carried out with iQ SYBR Green Supermix (Mei5 Bioservices Co., Ltd, Beijing, China). All primers for qPCR were supplied by Sangon Biotech (Shanghai, China). Relative expression levels were quantified using the 2^−ΔΔCt^ method. The sequences were as follows:

*Atf4*-Forward: 5′-CCTGAACAGCGAAGTGTTGG-3′;
*Atf4*-Reverse: 5′-TGGAGAACCCATGAGGTTTCAA-3′;
*Atf6*-Forward: 5′-AGCGCCCAAGACTCAAACC-3′;
*Atf6*-Reverse: 5′-CTGTATGCTGATAATCGACTGCT-3′;
*Xbp1*-Forward: 5′-GACAGAGAGTCAAACTAACGTGG-3′;
*Xbp1*-Reverse: 5′-GTCCAGCAGGCAAGAAGGT-3′;
*sXbp1*-Forward: 5′-AAACAGAGTAGCAGCTCAGACTGC-3′;
*sXbp1*-Reverse: 5′-TCCTTCTGGGTAGACCTCTGGGAG-3′;
*Myc*-Forward: 5′-TTCATCTGCGATCCTGACGAC-3′;
*Myc*-Reverse: 5′-CACTGAGGGGTCAATGCACTC-3′.

### 4.9. Western Blotting Analysis

PAGE Gel Fast Preparation Kit (7.5–12%) was purchased from EpiZyme (Shanghai, China). Polyvinylidene fluoride membranes (0.45 μm and 0.2 μm) were purchased from GE (Chicago, IL, USA). The Western blotting equipment was provided by Bio-Rad (Hercules, CA, USA). The following primary antibodies (CyclinD1, GRP78, P-PERK, PERK, CHOP, ATF4, and c-Myc) were purchased from Zenbio (Chengdu, China). Cleaved-caspase3 was purchased from Wanleibio (Shenyang, China). PCNA was purchased from Elabscience (Wuhan, China). TSC1, TSC2, P70S6K, and p-P70S6K were obtained from Proteintech (Wuhan, China). H3 and H3K27me3 were obtained from Solarbio (Beijing, China). Tubulin was obtained from Servicebio (Wuhan, China). Secondary antibodies were purchased from Proteintech. Primary antibodies (1:1000) were applied overnight at 4 °C, while secondary antibodies (1:2500) underwent a 1.5 h incubation at room temperature. The membrane was scanned using Amersham Imager 600, and images were analyzed using ImageJ.

### 4.10. Immunofluorescence Staining and Confocal Imaging

We treated the cells according to the following protocol: fixation in 4% paraformaldehyde (PFA, Solarbio, 30 min), permeabilization in 0.5% Triton X-100 (15 min) at room temperature, and blocking in 1% bovine serum albumin (BSA, Solarbio, 1 h, 37 °C). The cells were then incubated with primary antibodies (overnight, 4 °C) and corresponding secondary antibodies (2 h, room temperature). Following nuclear staining with 4′,6-Diamidino-2-Phenylindole (DAPI, Solarbio, 15 min), cell images were captured using a Zeiss laser scanning confocal microscope.

### 4.11. Measurement of Blood Urea Nitrogen (BUN) and Creatinine (CRE) Levels

Serum concentrations of BUN and CRE were quantified with assay kits (Nanjing Jiancheng Bioengineering Institute, Nanjing, China), strictly following the provided protocol.

### 4.12. In Vivo Animal Models

*Tsc1^+/−^* and *Tsc2^+/−^* mice on a C57BL/6 background were used in this study. For each genotype, 12–13-month-old male mice were randomly divided into four treatment groups (*n* = 3 per group): control, GSK-J4 (0.4 mg/kg, subcutaneous injection), rapamycin (1 mg/kg, intraperitoneal injection), and a combination of GSK-J4 and rapamycin. GSK-J4 was administered daily for 10 consecutive days, while rapamycin was given every other day over a 20-day period. After the final treatment, all mice were euthanized, and blood samples were obtained via retro-orbital bleeding. Serum was separated from each sample by centrifugation at 3000× *g* for 5 min at 4 °C. Data are presented as mean ± SD. Comparisons among multiple groups were analyzed by one-way ANOVA. A *p*-value of less than 0.05 was considered statistically significant.

### 4.13. Statistical Analysis

GraphPad Prism software (version 9.5.0) was utilized for data analysis and image generation. Data are expressed as mean ± SD. The *t*-test was employed for comparisons between two groups, and one-way ANOVA was used for multiple group comparisons. Values of *p* < 0.05 were considered statistically significant.

## 5. Conclusions

This study elucidates that GSK-J4 suppresses proliferation and induces apoptosis in primary *Tsc1^+/−^* and *Tsc2^+/−^* MEFs via activation of the ERS pathway. Moreover, GSK-J4 downregulates the proto-oncogene c-Myc through the ER stress-dependent PERK activation. GSK-J4 exhibits synergy with rapamycin in inhibiting cell growth. Meanwhile, the combination ameliorates renal impairment in *Tsc1-* or *Tsc2*-deficient models *in vivo*. These findings indicate that co-administration of GSK-J4 and rapamycin may represent a promising therapeutic strategy for TSC.

## Data Availability

The data supporting the conclusions of this article are included within the article and its Supplementary Information.

## Supplementary Materials

The following supporting information can be downloaded at: https://www.mdpi.com/article/10.3390/ijms27073067/s1.

## Abbreviations

The following abbreviations are used in this manuscript:

TSC	tuberous sclerosis complex
MEFs	mouse embryonic fibroblasts
ERS	endoplasmic reticulum stress
MDS	myelodysplastic syndromes
AML	acute myeloid leukemia
FBS	fetal bovine serum
DPBS	Dulbecco’s phosphate-buffered saline
EDTA	Ethylenediaminetetraacetic acid
PFA	paraformaldehyde
DAPI	4′,6-Diamidino-2-Phenylindole
BUN	blood urea nitrogen
DMEM	Dulbecco’s Modified Eagle Medium
CRE	blood creatinine
UPR	unfolded protein response
TGCT	testicular germ cell tumor
4-PBA	4-phenylbutyric acid
mTORC1	mammalian target of rapamycin complex 1
PRC2	Polycomb Repressive Complex 2
IL-1β	interleukin-1β
