# Supplementary material for: GSK-J4 Suppresses Tumorigenesis by Targeting the PERK-c-Myc Pathway Through Endoplasmic Reticulum Stress Activation in Tuberous Sclerosis Complex
Source: Int J Mol Sci. 2026 Mar 27;27(7):3067. doi: 10.3390/ijms27073067 (PMC13073512; doi:10.3390/ijms27073067)
Supplement: Supplementary file 1 [file ijms-27-03067-s001.zip › ijms-4206992-supplementary.pdf]

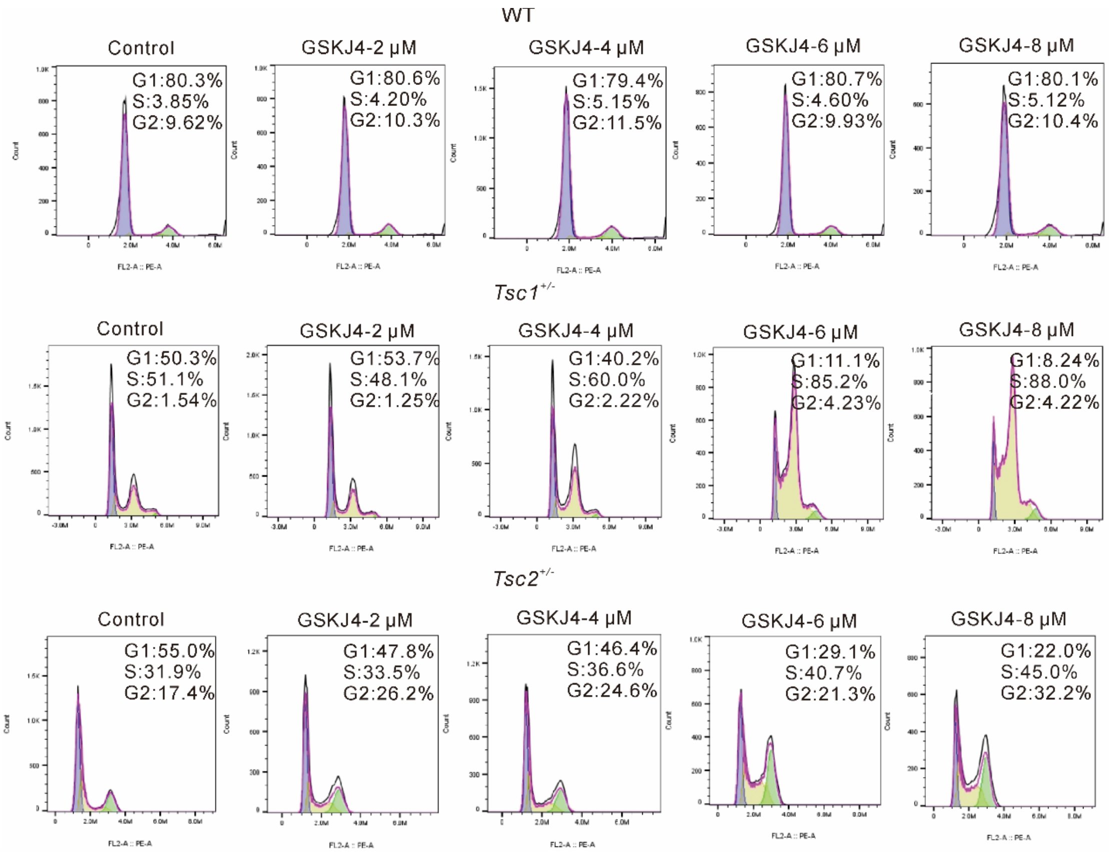

Supplementary Figure S1. Representative flow cytometry histograms showing cell cycle distribution in primary MEFs (WT, *Tsc1*$^{+/-}$, *Tsc2*$^{+/-}$) treated with GSK-J4 at its different concentrations for 48 hours.

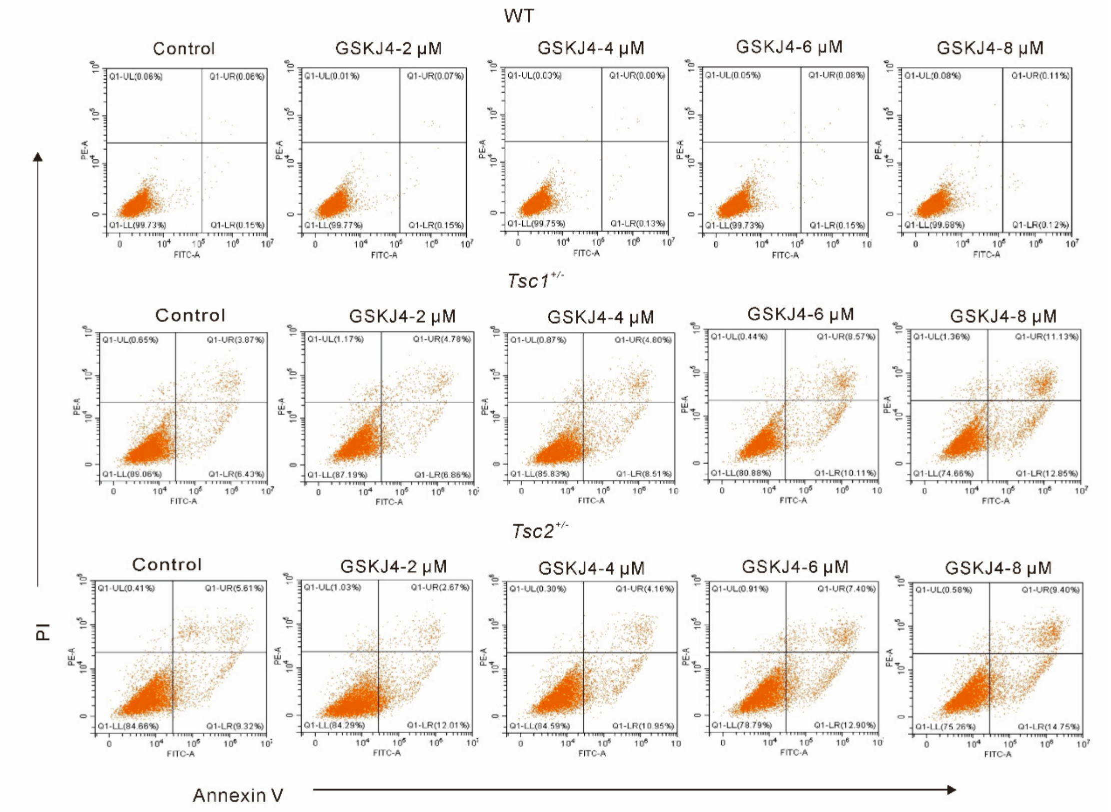

Supplementary Figure S2. Representative histograms of flow cytometric analysis for apoptosis in primary WT, *Tsc1*$^{+/-}$ and *Tsc2*$^{+/-}$ MEFs following 48-hour treatment with varying concentrations of GSK-J4.

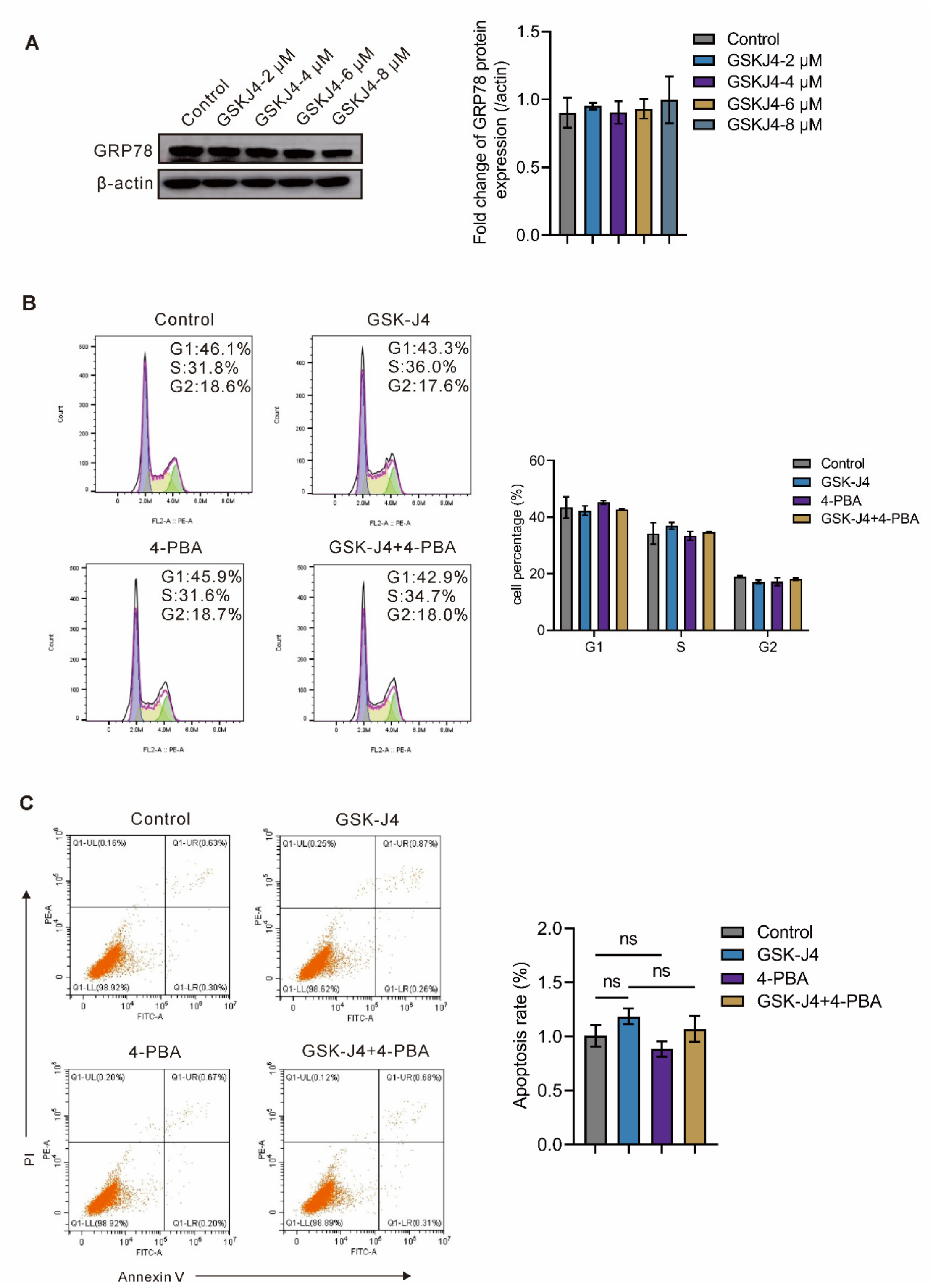

Supplementary Figure S3. (A) The expression of GRP78 in WT MEFs treated with different concentrations of GSK-J4 for 48h detected by Western blotting. (B) The cell cycle distribution in WT MEFs treated with 4-PBA combined with GSK-J4 (3 mM 4-PBA+ 6 µM GSK-J4) for 48h detected by flow cytometry. (C) Cell apoptosis analysis in WT MEFs treated with 4-PBA combined with GSK-J4 (3 mM 4-PBA+ 6 µM GSK-J4) for 48h detected by flow cytometry. ns, not significant.

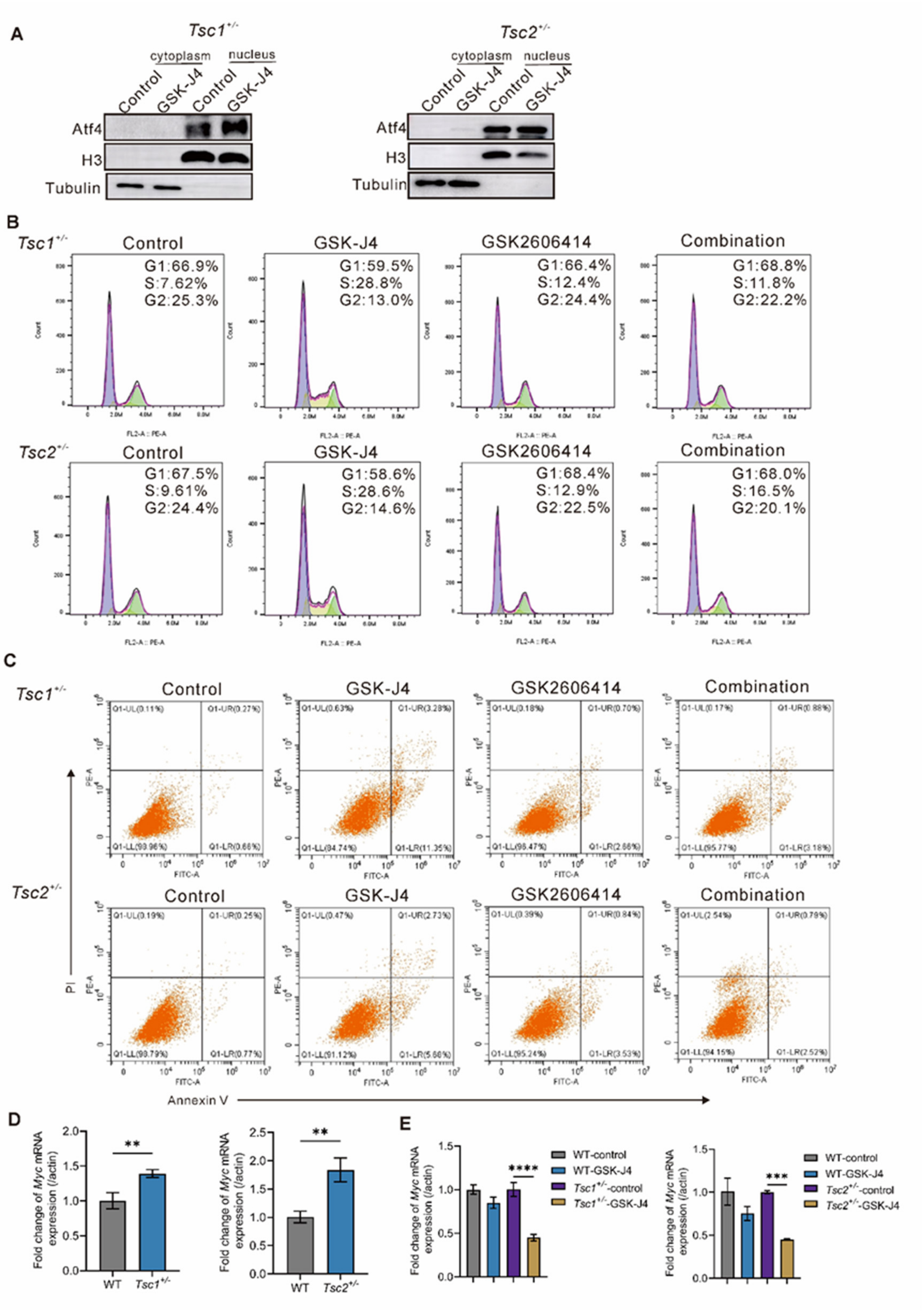

Supplementary Figure S4. (A) The cellular localization of ATF4 in *Tsc1*$^{+/-}$ and *Tsc2*$^{+/-}$ MEFs treated with GSK-J4 (6 μM) determined through nuclear and cytoplasmic protein extraction experiment. (B) Representative flow cytometry histograms showing cell cycle distribution in each group after drug treatment for *Tsc1*$^{+/-}$ and *Tsc2*$^{+/-}$ MEFs [Control, GSK-J4 (6 μM), GSK2606414 (10 μM), GSK-J4 (6 μM)+GSK2606414 (10 μM)]. (C) Representative histograms of flow cytometric

analysis for apoptosis in each group after drug treatment for *Tsc1*$^{+/-}$ and *Tsc2*$^{+/-}$ MEFs [Control, GSK-J4 (6 μM), GSK2606414 (10 μM), GSK-J4 (6 μM)+GSK2606414 (10 μM)]. (D) The mRNA levels of c-Myc in primary WT, *Tsc1*$^{+/-}$ and *Tsc2*$^{+/-}$ MEFs detected by qPCR. (E) The mRNA levels of c-Myc in primary WT, *Tsc1*$^{+/-}$ and *Tsc2*$^{+/-}$ MEFs treated with GSK-J4 (6 μM) detected by qPCR. $^{**}p < 0.01$, $^{***}p < 0.001$, $^{****}p < 0.0001$.

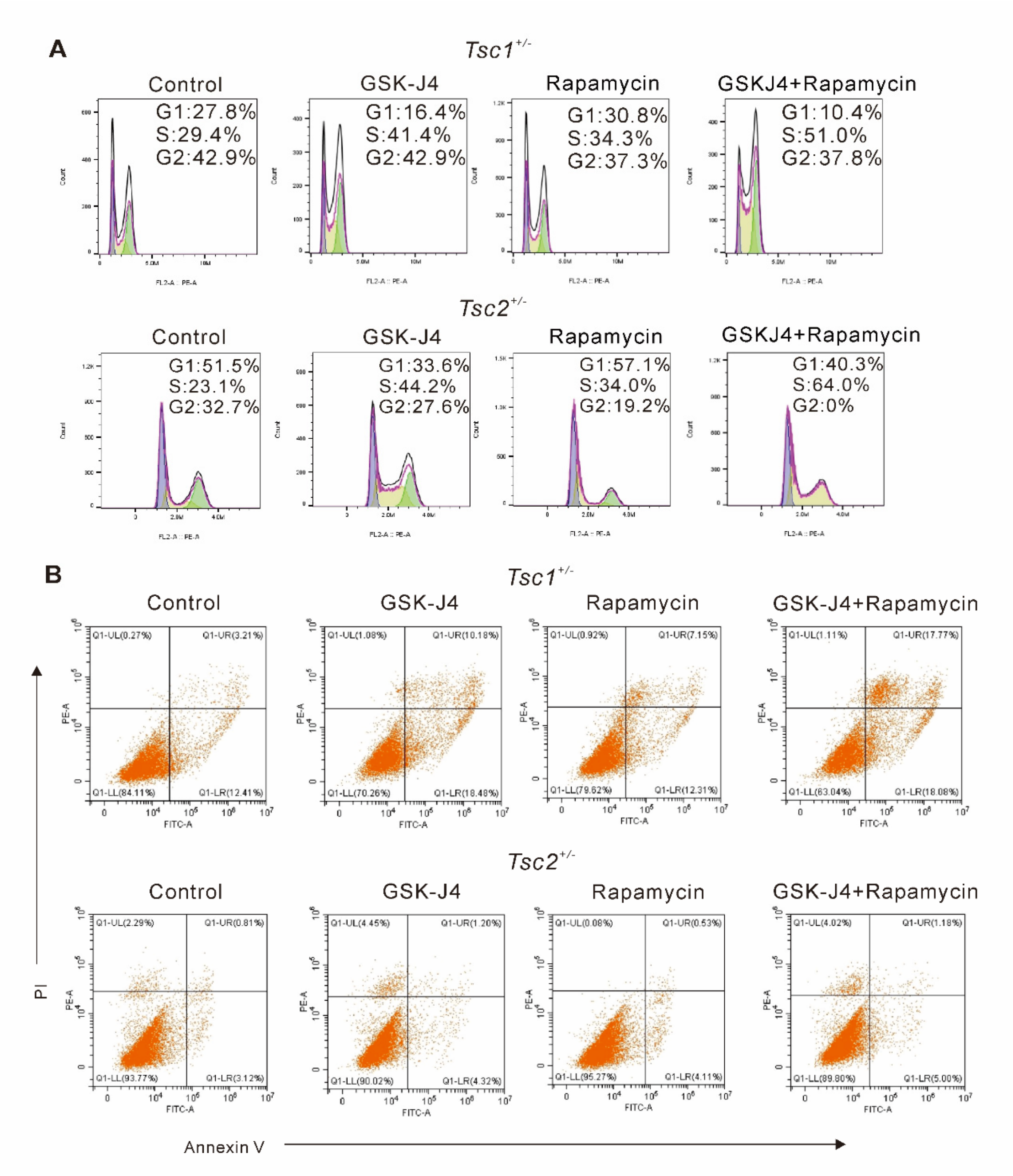

Supplementary Figure S5. (A) Representative flow cytometry histograms showing cell cycle distribution in cells (primary *Tsc1*$^{+/-}$ and *Tsc2*$^{+/-}$ MEFs) treated with the GSK-J4 (2 μM) and rapamycin (100 nM), individually and in combination. (B) Representative histograms of flow cytometric analysis for apoptosis in primary *Tsc1*$^{+/-}$ and *Tsc2*$^{+/-}$ MEFs treated with the GSK- J4 (2 μM) and rapamycin (100 nM), individually and in combination.

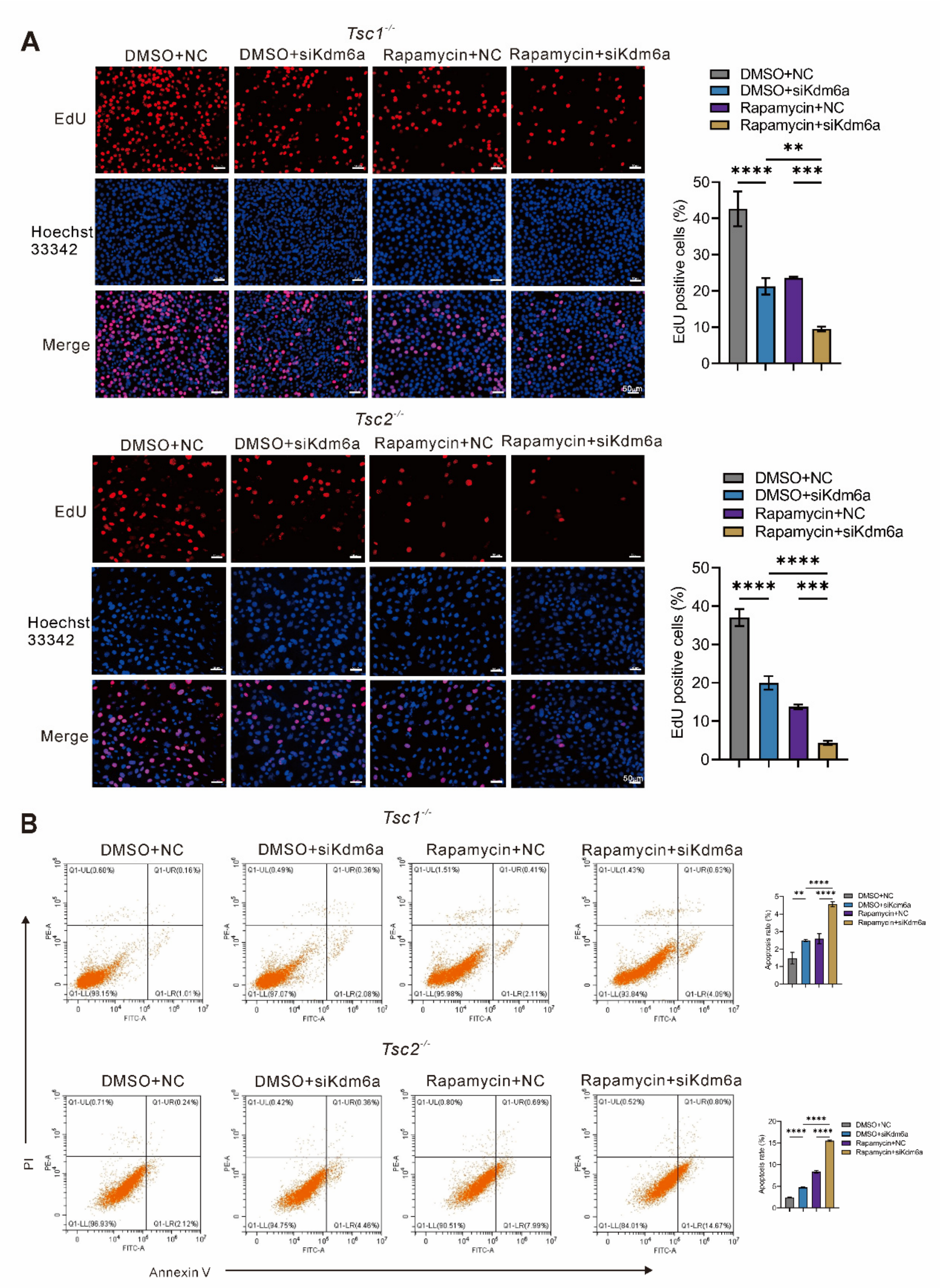

Supplementary Figure S6. Effects of *Kdm6a* knockdown on rapamycin-mediated cell proliferation and apoptosis in *Tsc1*$^{-/-}$ and *Tsc2*$^{-/-}$ MEFs. (A) EdU staining images, Scale bars = 50μm. (B) Cell apoptosis analyzed by flow cytometry. $^{**}p < 0.01$, $^{***}p < 0.001$, $^{****}p < 0.0001$.

	Body weight (g)	Liver weight (g)	Liver length (cm)	Liver width (cm)	Spleen length (cm)	Spleen width (cm)
WT	27.71±2.719	1.202±0.04	3.3	2.3	1.4	0.4
Tsc1$^{+/-}$	28.02±2.747	2.155±0.24**	3.6	2.1	1.7	0.5
Tsc2$^{+/-}$	27.78±3.041	4.667±0.13****	4.5	3.5	2.0	0.7

Supplementary Figure S7. Quantitative measurements of organ parameters. The table provided the numerical values of body weight, liver weight, liver length/width, and spleen length/width for WT, *Tsc1*$^{+/-}$ and *Tsc2*$^{+/-}$ mice. $^{**}p< 0.01$, $^{****}p< 0.0001$.
